# Supplementary material for: Predicting rhizosphere-competence-related catabolic gene clusters in plant-associated bacteria with rhizoSMASH
Source: Nat Commun. 2025 Sep 25;16:8400. doi: 10.1038/s41467-025-63526-8 (PMC12462448; doi:10.1038/s41467-025-63526-8)
Supplement: Supplementary file 1 — Supplementary Information [file 41467_2025_63526_MOESM1_ESM.pdf]

| Collection | # Acc | | Project Number | Ref |
|---|---|---|---|---|
| | Tot | Sub | | |
| REFSOIL | 842 | 842 | n. a. | 1 |
| RHIZATHA | 194 | 194 | PRJNA297942 | 2 |
| SOILATHA | 32 | 32 | PRJNA298127 | |
| RHIZHVUL | 46 | 41 | PRJEB42773 | 3 |
| | | 1 | PRJNA908138 | 4 |
| | | 1 | PRJNA507263 | 5 |
| | | 1 | PRJNA344944 | 6 |
| | | 2 | PRJNA67537, PRJNA67535 | 7 |
| RHIZOSAT | 45 | 20 | PRJNA701950 | 8 |
| | | 1 | PRJNA905546 | 9 |
| | | 1 | PRJNA880516 | 10 |
| | | 1 | PRJNA823515 | 11 |
| | | 1 | PRJNA821328 | 12 |
| | | 1 | PRJDB8374 | 13 |
| | | 1 | PRJNA531785 | 14 |
| | | 1 | PRJNA494794 | 15 |
| | | 1 | PRJNA454179 | 16 |
| | | 1 | PRJDB5588 | 17 |
| | | 1 | PRJNA414941 | 18 |
| | | 1 | PRJNA379921 | 19 |
| | | 1 | PRJNA352539 | 20 |
| | | 1 | PRJNA263863 | 21 |
| | | 1 | PRJNA168054 | 22 |
| | | 7 | PRJNA430105 | n. a. |
| | | 4 | PRJNA1047279, PRJNA942581, PRJNA597415, PRJNA544607 | |
| RHIZTAES | 48 | 11 | PRJNA667745 | 23 |
| | | 7 | PRJNA745065 | 24 |
| | | 3 | PRJNA643659 | 25 |
| | | 3 | PRJNA273703, PRJNA275697, PRJNA275699 | 26 |
| | | 3 | PRJNA67535, PRJNA67537, PRJNA67539 | 7 |
| | | 1 | PRJNA245780 | 27 |
| | | 1 | PRJNA309751 | 28 |
| | | 1 | PRJNA435479 | 29 |
| | | 1 | PRJNA488819 | 30 |
| | | 1 | PRJNA531142 | 31 |
| | | 1 | PRJNA623691 | 32 |
| | | 1 | PRJNA635904 | 33 |
| | | 1 | PRJNA71317 | 34 |
| | | 1 | PRJNA728132 | 35 |
| | | 1 | PRJNA741525 | 36 |
| | | 1 | PRJNA78839 | 37 |
| | | 1 | PRJNA824280 | 38 |
| | | 1 | PRJNA886615 | 39 |
| | | 1 | PRJNA605675 | 40 |
| | | 7 | PRJNA960949, PRJNA202956, PRJNA545192, PRJNA635401, PRJNA714046, PRJNA721340, PRJNA728357 | n. a. |
| RHIZSLYC | 21 | 3 | PRJEB19151, PRJEB19165, PRJEB19184 | 41 |
| | | 2 | PRJNA603418, PRJNA604468 | 42 |
| | | 1 | PRJNA239153 | 43 |
| | | 1 | PRJNA264437 | 44 |
| | | 1 | PRJNA780183 | 45 |
| | | 1 | PRJNA795281 | 46 |
| | | 1 | PRJNA940804 | 47 |
| | | 1 | PRJNA509704 | 48 |
| | | 1 | PRJNA531642 | 49 |
| | | 2 | PRJNA397822 | n. a. |
| | | 7 | PRJNA602778, PRJNA602780, PRJNA264433, PRJNA264436, PRJNA646039, PRJNA695080, PRJNA993421 | |

**Supplementary Table 1.** References and collection sizes of subcollections in the BARS genome collection. An n. a. mark in the reference column means the genomes were only documented sunder project numbers.

| Substrate Category | rCGC name | Evidence Levels | | | |
|---|---|---|---|---|---|
| | | Genetic | Expression | Ecological | Catabolic |
| Carbohydrate | Xylose | 50 | 50–52 | 50,53,54 | 50,51,54 |
| | Fructose PTS | | 51,52 | | 51 |
| | Mannitol | | 51,55,56 | 50 | 51,57 |
| | Inositol | 58,59 | 51,55,56,58,60 | 58,61 | 51,62 |
| | Trehalose PTS | | | 54 | 51,54,63 |
| | Trehalose trehalase | 59,64 | | | |
| | Alpha-Diglucoside phosphorylase | | | | 51,63 |
| | Sucrose levan-detour | 65 | | 54 | 51,54,63,65 |
| | Sucrose phosphorylase | 59 | | | |
| | Sucrose hydrolase | | | | |
| | Xylan | | 55 | | 50 |
| Organic acid | Glutarate dioxygenase | | | | 66,67 |
| | Glutarate succinyl-CoA | | | | |
| | Threonate Erythronate | | | | 66,68 |
| | 3-Oxoadipate | 69 | 51 | | * |
| | 2-Hydroxypentadienoate | | 51 | | |
| Amino acid | L-Proline | | 70 | | 51,63 |
| | D-Proline | | | | 63,71 |
| | LysArgOrn decarboxylase | | 56 | | 51,63,68,72 |
| | L-Lysine monooxygenase | | | | |
| | L-Lysine e-aminotransferase | | | | |
| | D-Lysine aminoadipate | | | | 54 |
| | Glutamine synthetase | | 55 | | 51,63,73 |
| | Glutamate synthase | | 51,55,74 | | |
| | Glutaminae | | | | |
| | Glutamate dehydrogenase | | | | |
| | GABA/AMV | 75 | 51,75 | | 51,66 |
| Amine | Monoamine quinohemoprotein dh | | | | 54 |
| | Monoamine Cu-dependent oxidase | | | 54 | |
| | Tyramine/Dopamine | | | 54 | 54 |
| | Polyamine gama-glutamylation | 76,77 | 51 | 54 | 54 |
| | Agmatine | | 51 | | |
| Phytohormone | IAA-biosyn IAM | 78,79 | | 54,80 | 4,51,81–83 |
| | IAA-biosyn IPyA | 84 | 85,86 | 54,80 | |
| | IAA-biosyn IAN | | | 54,80 | |
| | Phenylacetate | | 51,74 | 54,87 | |
| | IAA-degrad iac | 88 | 89 | | 63,66,90 |
| | IAA-degrad iad | | 91 | 91 | |
| | IAA-degrad ian | | | 87 | |
| | Salicylate 1-hydroxylase | | | | 63,66,92,93 |
| | Salicylate 5-hydroxylase | 94 | 94 | 87 | |
| | Salicylate CoA-ligase | | | | |
| Aromatic | Catechol ortho | | 51 | | ** 54,63 |
| | Protocatechuate ortho | 69 | 51,95 | | |
| | Protocatechuate para | | 51 | | |
| | Catechol meta | | | | |
| | Gentisate | | | | |
| | Benzoate | 69 | 51 | 87 | |
| | Anthranilate | | 51 | | |
| | Naphthalene | | | | 96 |
| | Vanillate | | | | 63 |
| | Quinate/Shikimate | | | | 51,63,66 |
| | Xanthine | | | | 63 |
| | Nicotinate | | | | 63,66 |

**Supplementary Table 2.** Substrate categories, evidence level and references of rCGC detection rules (strict rules, n=54) in the working version of rhizoSMASH. Gene clusters marked with * and ** are downstream and central pathways for aromatic compound catabolism.

| GenBank Acc. | Species | Strain | Isolated From |
|---|---|---|---|
| GCA_000021925.1 | *Desulfitobacterium hafniense* | DCB-2 | Municipal sludge, Denmark |
| GCA_000010045.1 | | Y51 | Soil contaminated with tetrachloroethene in Japan |
| GCA_000243155.3 | *Desulfitobacterium dehalogenans* | ATCC 51507 | Sediment in freshwater pond, Athens, GA |
| GCA_000243135.3 | *Desulfitobacterium dichloroeliminans* | LMG P-21439 | Soil matrix of an anoxic water-saturated layer (1 m in depth) that had been exclusively polluted with 1,2-DCA |
| GCA_000235605.1 | *Desulfosporosinus orientis* | DSM 765 | Soil at pumping station near rising main Rangoon Road |
| GCA_000255115.3 | *Desulfosporosinus acidiphilus* | SJ4 | Acid mining effluent decantation pond |
| GCA_000231385.3 | *Desulfosporosinus meridiei* | DSM 13257 | Groundwater contaminated with aromatic compounds from motor fuel in sandy soil from Eden Hill, Swan Coastal Plain, Perth, Western Australia |
| GCA_000512895.1 | *Dehalobacter restrictus* | DSM 9455 | PCE-dechlorinating packed-bed column; The Netherlands |
| GCA_000027145.1 | *Listeria seeligeri* | SLCC3954 | Soil, Germany |
| GCA_000008285.1 | *Listeria monocytogenes* | F2365 | Cheese product that caused an outbreak of listeriosis among patients with AIDS in California in 1985 |
| GCA_000168635.2 | | J0161 | A deli in the United States in 2000 during an epidemic outbreak |
| GCA_000022925.1 | | 08-5923 | Blood, clinical isolate during a nationwide outbreak |
| GCA_000093125.2 | | 08-5578 | Blood, reference outbreak strain during a national outbreak |
| GCA_000209755.1 | | L99 | Cheese; Netherlands |
| GCA_000021185.1 | | HCC23 | Channel catfish |
| GCA_000218305.1 | | M7 | Cow's milk; China |
| GCA_000210815.2 | | SLCC2372 | spinal fluid of man with cerebrospinal meningitis |
| GCA_000307615.1 | | SLCC2378 | Poultry |
| GCA_000195795.1 | *Listeria innocua* | Clip11262 | Dairy products (cheese) from Morocco |
| GCA_000210795.2 | *Listeria monocytogenes* | SLCC2482 | Human |
| GCA_000091785.1 | *Legionella longbeachae* | NSW150 | Human |
| GCA_000092545.1 | *Legionella pneumophila* | Corby | Human |
| GCA_000048645.1 | | Paris | Human, endemic in France |
| GCA_000239175.1 | | ATCC 43290 | Human lung tissue; USA |
| GCA_000092625.1 | | 2300/99 Alcoy | Patient affected by legionellosis in Alcoy (Spain) |
| GCA_000048665.1 | | Lens | Human; France |
| GCA_000250675.3 | *Nocardia brasiliensis* | ATCC 700358 | Human mycetoma, Monterrey, Mexico |
| GCA_000284035.1 | *Nocardia cyriacigeorgica* | GUH-2 | Human nocardiosis case |
| GCA_000009805.1 | *Nocardia farcinica* | IFM 10152 | Bronchus of a 68-year-old male Japanese patient |
| GCA_000523235.1 | *Nocardia nova* | SH22a | Root of *Couma macrocarpa* in Brazil |
| GCA_000454045.1 | *Rhodococcus erythropolis* | CCM2595 | Soil |
| GCA_000014565.1 | *Rhodococcus jostii* | RHA1 | Soil contaminated with gamma-hexachlorocyclohexane in Japan |
| GCA_000599545.1 | *Rhodococcus opacus* | PD630 | Soil sample in Germany |
| GCA_003641205.1 | *Rhodococcus sp.* | P1Y | Rhizosphere of rice; Russia |

**Supplementary Table 3.** The environmental origins of bacterial strains from families *Desulfitobacteriaceae*, *Listeriaceae*, *Legionellaceae* and *Nocardiaceae*. We searched the JGI GOLD database with GenBank assembly accession number and full strain names in the corresponding families and summarized "sample collection site", "isolation country/ocean", "ecosystem category" and "ecosystem type" fields from the results, combined with their annotations on NCBI. Strains without detailed annotations for these fields were excluded from the table.

| Dataset | Host | Predictor Type | Sensitivity | Specificity | Precision |
|---|---|---|---|---|---|
| Zboralski et al. | *Arabidopsis* | Catabolism-assay | 0.87 | 0.80 | 0.90 |
| | | rCGC | 0.90 | 0.85 | 0.92 |
| | Potato | Catabolism-assay | 0.86 | 0.19 | 0.75 |
| | | rCGC | 0.82 | 0.69 | 0.88 |
| Zhalnina at al. | *A. barbata* | Catabolism-assay | 0.81 | 0.04 | 0.65 |
| | | rCGC | 0.89 | 0.37 | 0.77 |

**Supplementary Table 4**. Performance metrics (sensitivity, specificity and precision) of models in our study.

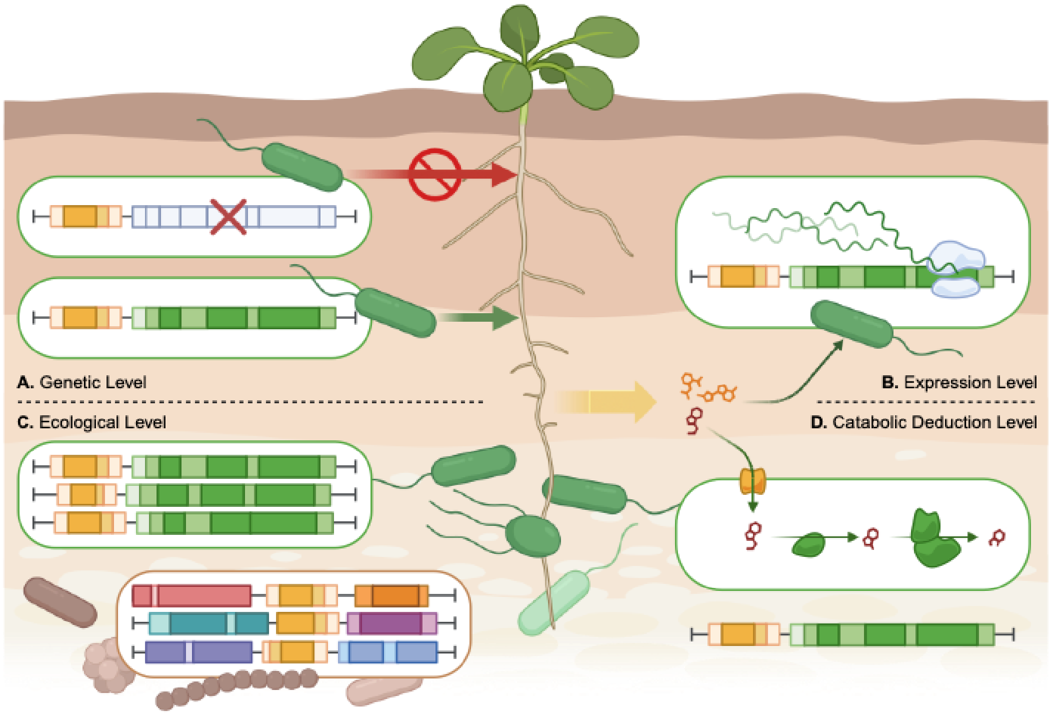


**Supplementary Figure 1.** A schematic representation showing the four evidence levels in our literature-based discoveries of known rCGCs. **A.** genetic level: interruption of the gene cluster directly affects rhizosphere competence; **B.** expression level, treatment of root exudates (or root exudate components) induces the expression of the gene cluster; **C.** ecological level, the gene cluster is enriched in the genomes of rhizosphere associated bacteria; **D.** catabolic deduction level, the gene cluster encodes a pathway to catabolize metabolites that are highly abundant in root exudates or are often utilized by rhizosphere associated bacteria.

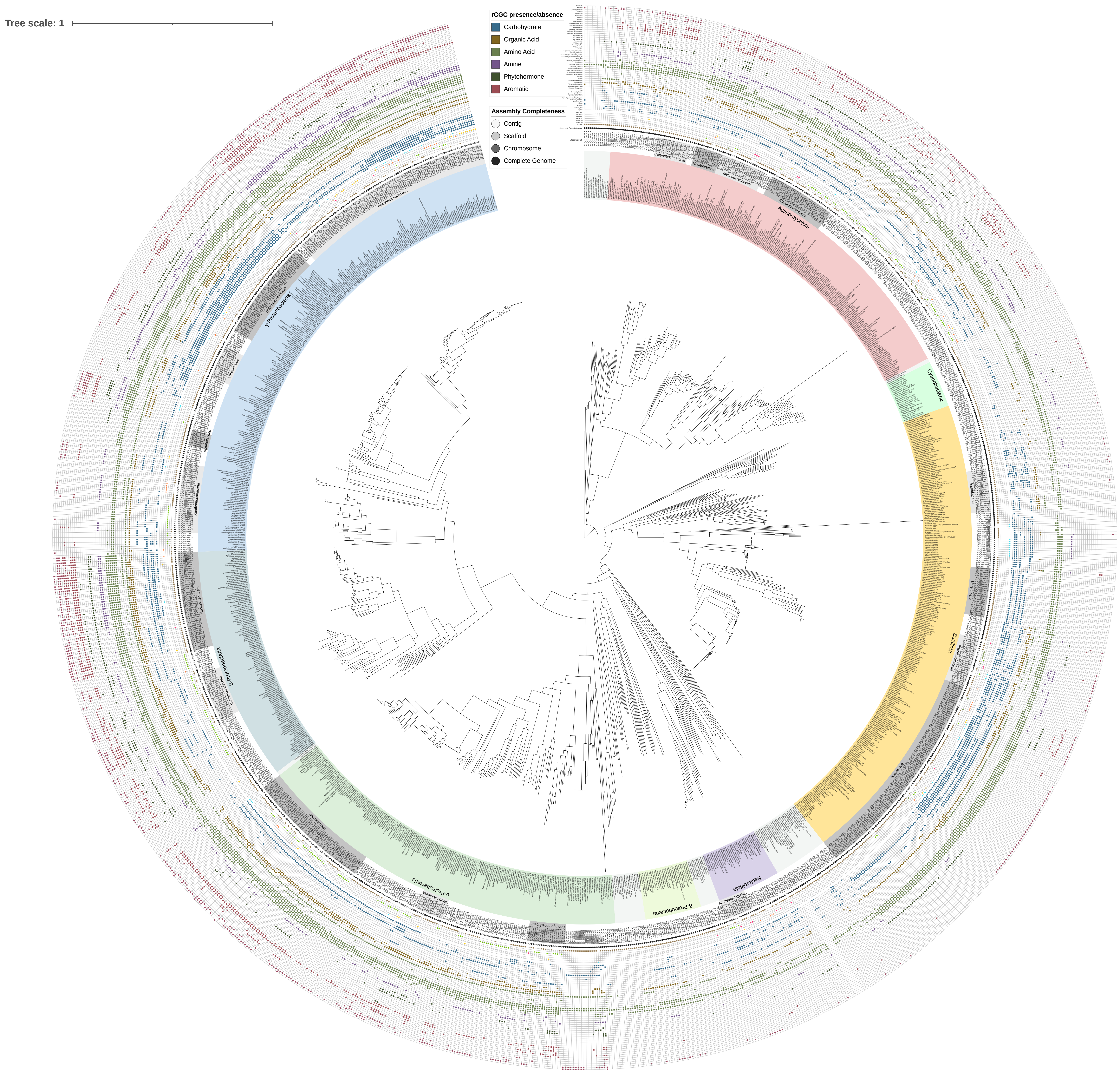

Tree scale: 1
rCGC presence/absence
Carbohydrate
Organic Acid
Amino Acid
Amine
Phytohormone
Aromatic
Assembly Completeness
Contig
Scaffold
Chromosome
Complete Genome
Actinomycetota
Cyanobacteria
Bacillota
Bacteroidota
δ-Proteobacteria
α-Proteobacteria
β-Proteobacteria
γ-Proteobacteria

**Supplementary Figure 2.** Distribution of rCGC presence and absence throughout all BARS genomes. The internal phylogenetic tree was constructed with GTDB-tk and rooted using the phylum *Chloroflexota*. Besides the leaves of the phylogenetic tree, we displayed the assembly completeness, the BARS subcollection and the rCGC presence/absence profile for each genome correspondingly. Colored ranges in the strain names indicates several major clades in BARS. Gray shaded ranges in the assembly IDs indicate the bacterial families used in **Figure 2a** of the main text.

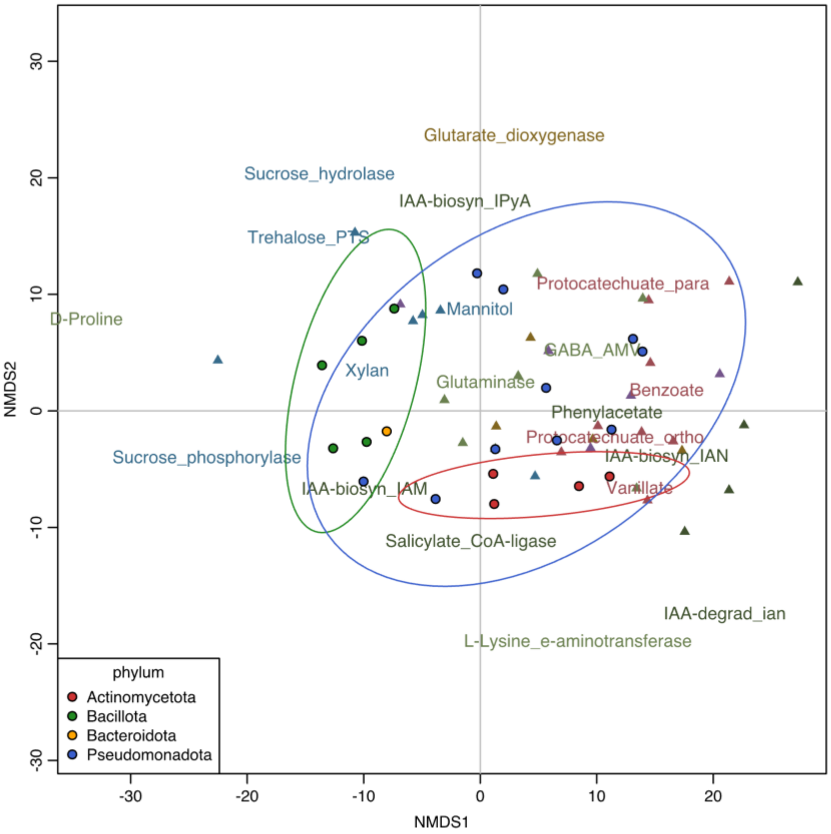


**Supplementary Figure 3.** NMDS of bacterial families according to the prevalence of each rCGC type. Each point on the plot stands for a family in Figure 2 in the main text. The color of a point represents its phylum. Phyla of these families are grouped with ellipses, representing the 95% confidence interval of the group. Text labels and triangles represents rCGC types, with the same color scheme as in Figure 2.

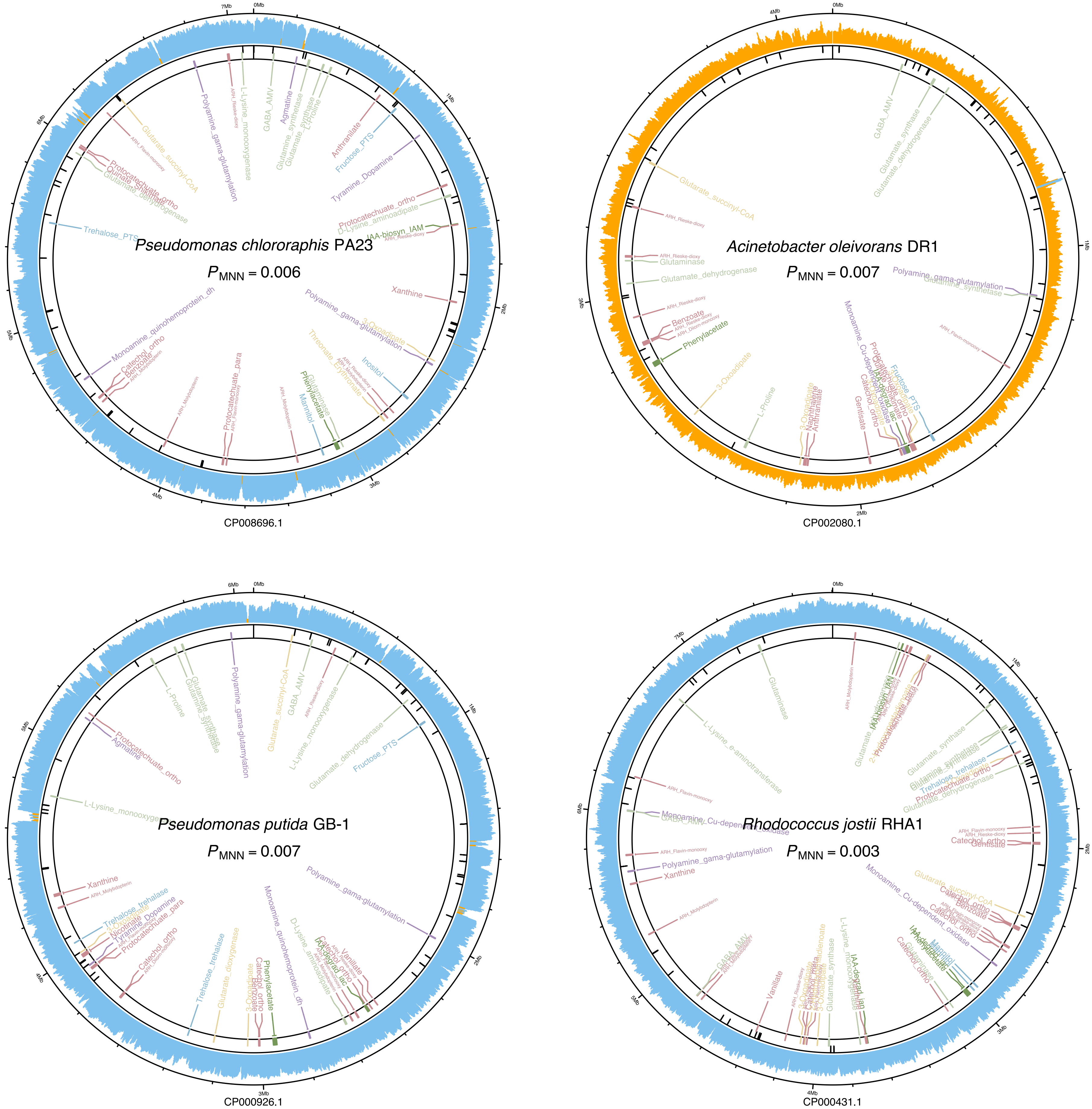

Pseudomonas chlororaphis PA23
$P_{MNN}$ = 0.006
CP008696.1
Acinetobacter oleivorans DR1
$P_{MNN}$ = 0.007
CP002080.1
Pseudomonas putida GB-1
$P_{MNN}$ = 0.007
CP000926.1
Rhodococcus jostii RHA1
$P_{MNN}$ = 0.003
CP000431.1

**Supplementary Figure 4.** Distribution of rCGCs on the chromosome of four example bacterial strains. The outer ring shows the GC content (with a sliding window of 5 kbps and step of 2 kbps), where blue and orange indicate if local GC content is above 0.5 or not. Black sticks on the middle ring depict the positions of tRNA genes (normally found at the boundaries of genomic islands). The colored sticks on the inner ring indicate the positions of rCGCs, where the colors follow the same scheme in Figure 2. Text labels inside the ring are the name of rCGCs, where smaller text labels are relaxed rules (for the working version, those are core gene clusters encoding the aromatic ring hydroxylating enzymes). Relaxed rCGCs inside the region of other rCGCs are merged into the including rCGC. For each genome, a Monte Carlo simulation of the mean nearest neighborhood distance (MNN) of a uniform distribution was applied to test whether the rCGCs were evenly distributed or not ($P_{\text{MNN}}$ values under the name of the strain).

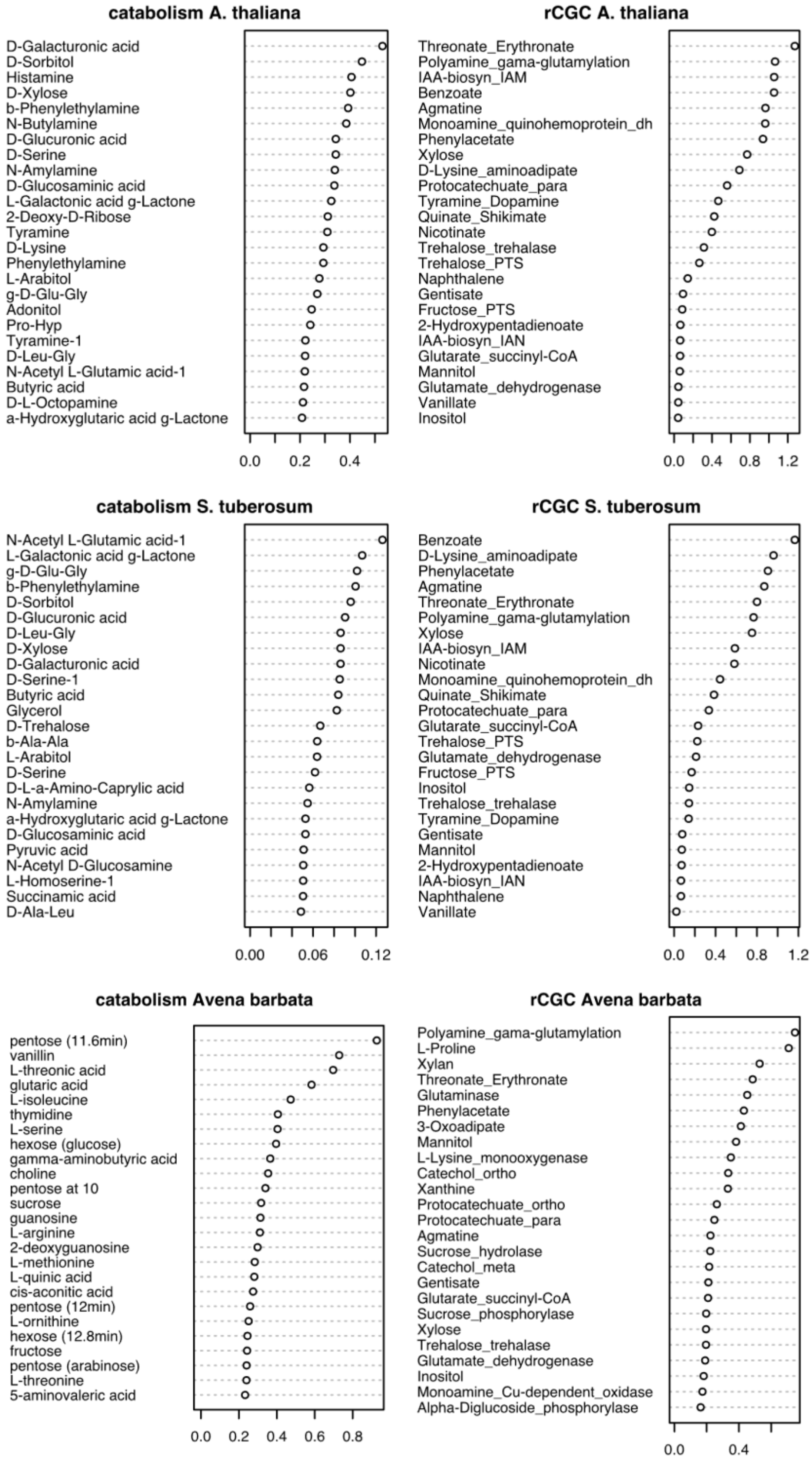


**Supplementary Figure 5.** Variable importance charts for random forest models predicting rhizosphere competence phenotypes in different plants and with different datasets. For each model, only the top 25 important predictors are listed on the charts.

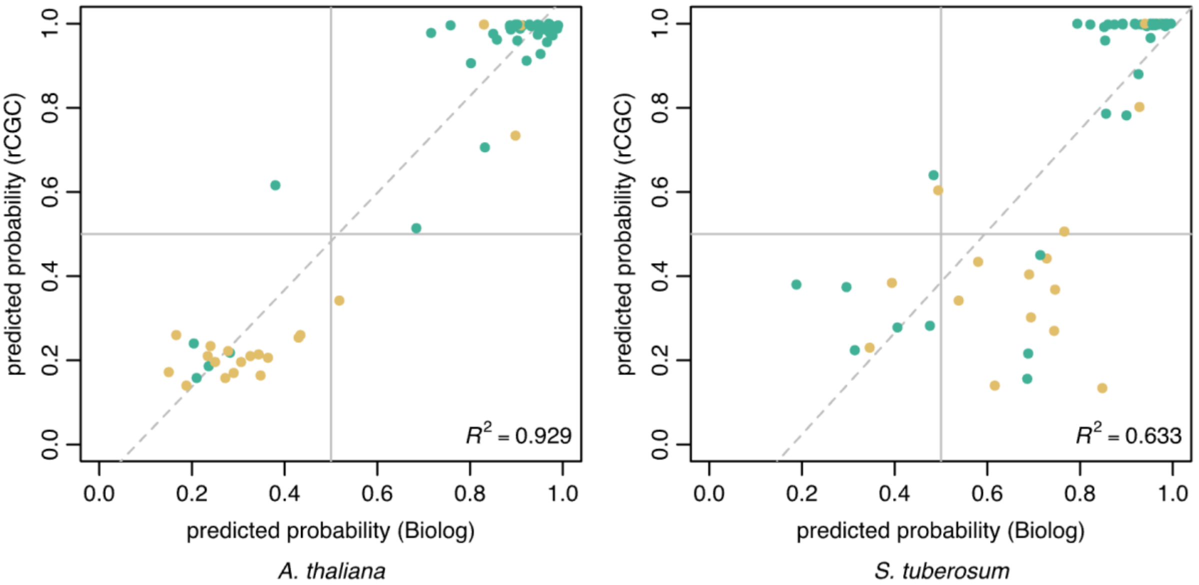


**Supplementary Figure 6**. Consistency of prediction between Biolog-assay-based and rCGC-based models in the first case study. The color of each point represents the reference rhizosphere colonization level (turquoise: med-high, yellow: low). The x-axis and y-axis positions of a point indicate the predicted probabilities (of being "med-high" in colonization) of the Biolog-assay-based model and the rCGC-based model respectively. The dashed line in each graph indicates the simple linear regression line. The $R^2$ value for each regression is denoted at the bottom right corner of each graph.

## References


1. Choi, J. *et al.* Strategies to improve reference databases for soil microbiomes. *ISME Journal* **11**, 829–834 (2017).

2. Bai, Y. *et al.* Functional overlap of the Arabidopsis leaf and root microbiota. *Nature 2015 528:7582* **528**, 364–369 (2015).

3. Robertson-Albertyn, S. *et al.* Genome-Annotated Bacterial Collection of the Barley Rhizosphere Microbiota. *Microbiol Resour Announc* **11**, 1064–1085 (2022).

4. Xu, F. *et al.* Auxin-producing bacteria promote barley rhizosheath formation. *Nat Commun* **14**, 5800 (2023).

5. Alvarez, F. *et al.* Genome mining of Burkholderia ambifaria strain T16, a rhizobacterium able to produce antimicrobial compounds and degrade the mycotoxin fusaric acid. *World J Microbiol Biotechnol* **38**, (2022).

6. Aamot, H. U., Hofgaard, I. S. & Lysøe, E. Complete genome sequence of Luteibacter rhizovicinus strain LJ96T, isolated from the rhizosphere of barley (Hordeum vulgare L.) in Denmark. *Genom Data* **11**, 104–105 (2016).

7. Loper, J. E. *et al.* Comparative genomics of plant-associated Pseudomonas spp.: insights into diversity and inheritance of traits involved in multitrophic interactions. *PLoS Genet* **8**, (2012).

8. Bez, C., Covaceuszach, S., Bertani, I., Choudhary, K. S. & Venturi, V. LuxR Solos from Environmental Fluorescent Pseudomonads. *mSphere* **6**, (2021).

9. Kammanee, S. *et al.* Saccharopolyspora oryzae sp. nov., isolated from rhizosphere soil of the wild rice species Oryza rufipogon. *J Antibiot (Tokyo)* **76**, 658–664 (2023).

10. Song, J. *et al.* Complete Genome Sequence of Pseudomonas promysalinigenes Strain RL-WG26, a PGPR Isolated from the Rice Rhizosphere for Studying the Promotion of Plant Growth Under Salt Stress. *https://doi.org/10.1094/PHYTOFR-10-22-0102-A* **3**, 906–910 (2023).

11. Butdee, W., Muangham, S., Chonudomkul, D. & Duangmal, K. Streptomyces rhizoryzae sp. nov., isolated from paddy rhizosphere soil and formal proposal to reclassify Streptomyces albulus as a later heterotypic synonym of Streptomyces noursei. *Int J Syst Evol Microbiol* **73**, 005639 (2023).

12. Kamal Rafedzi, E. A. *et al.* Draft Genome Sequence of the Drought-Tolerant Plant Growth-Promoting Rhizobacterium Bacillus altitudinis UKM RB11, Isolated from Upland Paddy Rhizosphere. *Microbiol Resour Announc* **11**, (2022).

13. Khalifa, A. *et al.* Methylomagnum ishizawai gen. nov., sp. nov., a mesophilic type i methanotroph isolated from rice rhizosphere. *Int J Syst Evol Microbiol* **65**, 3527–3534 (2015).

14. Lin, S. Y., Hameed, A., Huang, H. I. & Young, C. C. Allorhizobium terrae sp. nov., isolated from paddy soil, and reclassification of Rhizobium oryziradicis (Zhao et al. 2017) as Allorhizobium oryziradicis comb. nov. *Int J Syst Evol Microbiol* **70**, 397–405 (2020).

15. Gogoleva, N. E. *et al.* Complete Genome Sequence of Abscisic Acid-Metabolizing Rhizobacterium Rhodococcus sp. Strain P1Y. *Microbiol Resour Announc* **8**, (2019).

16. Menon, R. R. *et al.* Sphingomonas pokkalii sp. nov., a novel plant associated rhizobacterium isolated from a saline tolerant pokkali rice and its draft genome analysis. *Syst Appl Microbiol* **42**, 334–342 (2019).

17. Lv, H., Sahin, N. & Tani, A. Isolation and genomic characterization of Novimethylophilus kurashikiensis gen. nov. sp. nov., a new lanthanide-dependent methylotrophic species of Methylophilaceae. *Environ Microbiol* **20**, 1204–1223 (2018).

18. Budiharjo, A., Jeong, H., Wulandari, D., Lee, S. & Ryu, C. M. Complete Genome Sequence of Bacillus altitudinis P-10, a Potential Bioprotectant against Xanthomonas oryzae pv. oryzae, Isolated from Rice Rhizosphere in Java, Indonesia. *Genome Announc* **5**, (2017).

19. Menon, R. R., Kumari, S., Viver, T. & Rameshkumar, N. Flavobacterium pokkalii sp. nov., a novel plant growth promoting native rhizobacteria isolated from pokkali rice grown in coastal saline affected agricultural regions of southern India, Kerala. *Microbiol Res* **240**, (2020).

20. Chandrashekar, B. S. *et al.* Bacillus velezensis (strains A6 & P42) as a potential biocontrol agent against Klebsiella variicola, a new causal agent of soft rot disease in carrot. *Lett Appl Microbiol* **76**, (2023).

21. Liu, Q. *et al.* Genome sequence of Pseudomonas parafulva CRS01-1, an antagonistic bacterium isolated from rice field. *J Biotechnol* **206**, 89–90 (2015).

22. Liu, J. *et al.* Genome sequence of the biocontrol agent Microbacterium barkeri strain 2011-R4. *J Bacteriol* **194**, 6666–6667 (2012).

23. Strafella, S. *et al.* Comparative Genomics and In Vitro Plant Growth Promotion and Biocontrol Traits of Lactic Acid Bacteria from the Wheat Rhizosphere. *Microorganisms* **9**, 1–18 (2020).

24. Liu, H. *et al.* Effective colonisation by a bacterial synthetic community promotes plant growth and alters soil microbial community. *Journal of Sustainable Agriculture and Environment* **1**, 30–42 (2022).

25. Bartoli, C. *et al.* Rhizobium leguminosarum symbiovar viciae strains are natural wheat endophytes that can stimulate root development. *Environ Microbiol* **24**, 5509–5523 (2022).

26. Tovi, N., Frenk, S., Hadar, Y. & Minz, D. Host specificity and spatial distribution preference of three Pseudomonas isolates. *Front Microbiol* **10**, 3263 (2019).

27. Pieterse, C. M. J. *et al.* Pseudomonas simiae WCS417: star track of a model beneficial rhizobacterium. *Plant and Soil 2020 461:1* **461**, 245–263 (2020).

28. Matilla, M. A., Drew, A., Udaondo, Z., Krell, T. & Salmond, G. P. C. Genome Sequence of Serratia plymuthica A153, a Model Rhizobacterium for the Investigation of the Synthesis and Regulation of Haterumalides, Zeamine, and Andrimid. *Genome Announc* **4**, (2016).

29. Bajpai, A., Singh, B., Joshi, S. & Johri, B. N. Production and Characterization of an Antifungal Compound from Pseudomonas protegens Strain W45. *Proceedings of the National Academy of Sciences India Section B - Biological Sciences* **88**, 1081–1089 (2018).

30. Wash, P. *et al.* Deciphering the genetics of antagonism and antimicrobial resistance in Bacillus velezensis HU-91 by whole genome analysis. *J King Saud Univ Sci* **35**, 102954 (2023).

31. Hoyos, H. A. V. *et al.* Streptomyces rhizosphaericola sp. nov., an actinobacterium isolated from the wheat rhizosphere. *Int J Syst Evol Microbiol* **69**, 2431–2439 (2019).

32. Zarvandi, S. *et al.* Draft Genome Sequence of Cyclic Lipopeptide Producer Pseudomonas sp. Strain SWRI103, Isolated from Wheat Rhizosphere. *Microbiol Resour Announc* **9**, (2020).

33. Sun, X., Xu, Y., Chen, L., Jin, X. & Ni, H. The salt-tolerant phenazine-1-carboxamide-producing bacterium Pseudomonas aeruginosa NF011 isolated from wheat rhizosphere soil in dry farmland

with antagonism against Fusarium graminearum. *Microbiol Res* **245**, 126673 (2021).

34. Brito, L. F. *et al.* Complete genome sequence of Paenibacillus riograndensis SBR5(T), a Gram-positive diazotrophic rhizobacterium. *J Biotechnol* **207**, 30–31 (2015).

35. Montoya-Martínez, A. C. *et al.* Biological Control Mechanisms of Bacillus cabrialesii subsp. tritici TSO2T against Fusarium languescens, the Causal Agent of Wilt in Jalapeño Peppers. *Horticulturae 2023, Vol. 9, Page 964* **9**, 964 (2023).

36. Díaz, M. *et al.* Agronomic efficiency and genome mining analysis of the wheat-biostimulant rhizospheric bacterium Pseudomonas pergaminensis sp. nov. strain 1008T. *Front Plant Sci* **13**, (2022).

37. He, P. *et al.* Genome sequence of the plant growth promoting strain Bacillus amyloliquefaciens subsp. plantarum B9601-Y2 and expression of mersacidin and other secondary metabolites. *J Biotechnol* **164**, 281–291 (2012).

38. Li, X. *et al.* Arthrobacter rhizosphaerae sp. nov., isolated from wheat rhizosphere. *Arch Microbiol* **204**, (2022).

39. Ali, A., Dindhoria, K. & Kumar, R. Acinetobacter oleivorans IRS14 alleviates cold stress in wheat by regulating physiological and biochemical factors. *J Appl Microbiol* **134**, (2023).

40. Wash, P. *et al.* Deciphering the genetics of antagonism and antimicrobial resistance in Bacillus velezensis HU-91 by whole genome analysis. *J King Saud Univ Sci* **35**, 102954 (2023).

41. Bouam, A., Armstrong, N., Levasseur, A. & Drancourt, M. Mycobacterium terramassiliense, Mycobacterium rhizamassiliense and Mycobacterium numidiamassiliense sp. nov., three new Mycobacterium simiae complex species cultured from plant roots. *Sci Rep* **8**, (2018).

42. Lee, S. A. *et al.* Paenibacillus lycopersici sp. nov. and Paenibacillus rhizovicinus sp. nov., isolated from the rhizosphere of tomato (Solanum lycopersicum). *J Microbiol* **58**, 832–840 (2020).

43. Krzyzanowska, D. M., Ossowicki, A. & Jafra, S. Genome Sequence of Pseudomonas sp. Strain P482, a Tomato Rhizosphere Isolate with Broad-Spectrum Antimicrobial Activity. *Genome Announc* **2**, (2014).

44. Jung, E. J. *et al.* Flavobacterium dauae sp. nov., isolated from rhizosphere soil of a tomato plant. *Int J Syst Evol Microbiol* **71**, (2021).

45. Dong, H., Gao, R., Dong, Y., Yao, Q. & Zhu, H. Bacillus velezensis RC116 Inhibits the Pathogens of Bacterial Wilt and Fusarium Wilt in Tomato with Multiple Biocontrol Traits. *Int J Mol Sci* **24**, (2023).

46. Cong, S., Li, J. Z., Xiong, Z. Z. & Wei, H. L. Diverse interactions of five core type III effectors from Ralstonia solanacearum with plants. *Journal of Genetics and Genomics* **50**, 341–352 (2023).

47. Quiroz-Morales, S. E. *et al.* Pseudomonas aeruginosa strains belonging to phylogroup 3 frequently exhibit an atypical quorum sensing response: the case of MAZ105, a tomato rhizosphere isolate. *Microbiology (N Y)* **169**, 001401 (2023).

48. Kim, S. J. *et al.* Chitinophaga rhizosphaerae sp. Nov., isolated from rhizosphere soil of a tomato plant. *Int J Syst Evol Microbiol* **67**, 3435–3439 (2017).

49. Ngo, H. T. T., Kook, M. C. & Yi, T. H. Pedobacter ureilyticus sp. nov., isolated from tomato rhizosphere soil. *Int J Syst Evol Microbiol* **65**, 1008–1014 (2015).

50. Liu, Y., Rainey, P. B. & Zhang, X.-X. Molecular mechanisms of xylose utilization by Pseudomonas

fluorescens: overlapping genetic responses to xylose, xylulose, ribose and mannitol. *Mol Microbiol* **98**, 553–570 (2015).

51. Mavrodi, O. V. *et al.* Root Exudates Alter the Expression of Diverse Metabolic, Transport, Regulatory, and Stress Response Genes in Rhizosphere Pseudomonas. *Front Microbiol* **12**, 651282 (2021).

52. Oyserman, B. O. *et al.* Disentangling the genetic basis of rhizosphere microbiome assembly in tomato. *Nat Commun* **13**, (2022).

53. Lopes, L. D. *et al.* Genome variations between rhizosphere and bulk soil ecotypes of a Pseudomonas koreensis population. *Environ Microbiol* **20**, 4401–4414 (2018).

54. Zboralski, A., Biessy, A., Savoie, M. C., Novinscak, A. & Filion, M. Metabolic and genomic traits of phytobeneficial phenazine-producing Pseudomonas spp. Are linked to rhizosphere colonization in Arabidopsis thaliana and Solanum tuberosum. *Appl Environ Microbiol* **86**, (2020).

55. Zhang, N. *et al.* Whole transcriptomic analysis of the plant-beneficial rhizobacterium Bacillus amyloliquefaciens SQR9 during enhanced biofilm formation regulated by maize root exudates. *BMC Genomics* **16**, (2015).

56. Xie, S. *et al.* Transcriptome profiling of Bacillus subtilis OKB105 in response to rice seedlings. *BMC Microbiol* **15**, (2015).

57. Patel, J. S. *et al.* A Plant Biostimulant from Ascophyllum nodosum Potentiates Plant Growth Promotion and Stress Protection Activity of Pseudomonas protegens CHA0. *Plants (Basel)* **12**, (2023).

58. Sánchez-Gil, J. J. *et al.* The conserved iol gene cluster in Pseudomonas is involved in rhizosphere competence. *Current Biology* (2023) doi:10.1016/J.CUB.2023.05.057.

59. Hamilton, C. D., Steidl, O. R., MacIntyre, A. M., Hendrich, C. G. & Allen, C. Ralstonia solanacearum depends on catabolism of myo-inositol, sucrose, and trehalose for virulence in an infection stage-dependent manner. *Molecular Plant-Microbe Interactions* **34**, (2021).

60. Fan, B. *et al.* Transcriptomic profiling of Bacillus amyloliquefaciens FZB42 in response to maize root exudates. *BMC Microbiol* **12**, 1–13 (2012).

61. Weber, M. & Fuchs, T. M. Metabolism in the Niche: a Large-Scale Genome-Based Survey Reveals Inositol Utilization To Be Widespread among Soil, Commensal, and Pathogenic Bacteria. *Microbiol Spectr* **10**, (2022).

62. O'Banion, B. S. *et al.* Plant myo-inositol transport influences bacterial colonization phenotypes. *Curr Biol* **33**, 3111-3124.e5 (2023).

63. McLaughlin, S., Zhalnina, K., Kosina, S., Northen, T. R. & Sasse, J. The core metabolome and root exudation dynamics of three phylogenetically distinct plant species. *Nat Commun* **14**, 1649 (2023).

64. Higo, A., Katoh, H., Ohmori, K., Ikeuchi, M. & Ohmori, M. The role of a gene cluster for trehalose metabolism in dehydration tolerance of the filamentous cyanobacterium Anabaena sp. PCC 7120. *Microbiology (N Y)* **152**, 979–987 (2006).

65. Tian, T. *et al.* Sucrose triggers a novel signaling cascade promoting Bacillus subtilis rhizosphere colonization. *ISME J* **15**, 2723–2737 (2021).

66. Zhalnina, K. *et al.* Dynamic root exudate chemistry and microbial substrate preferences drive patterns in rhizosphere microbial community assembly. *Nat Microbiol* **3**, 470–480 (2018).

67. Kim, D. R. *et al.* Glutamic acid reshapes the plant microbiota to protect plants against pathogens. *Microbiome* **9**, 1–18 (2021).

68. Wen, T. *et al.* Deciphering the mechanism of fungal pathogen-induced disease-suppressive soil. *New Phytologist* **238**, 2634–2650 (2023).

69. Ledger, T. *et al.* Aromatic compounds degradation plays a role in colonization of Arabidopsis thaliana and Acacia caven by Cupriavidus pinatubonensis JMP134. *Antonie van Leeuwenhoek, International Journal of General and Molecular Microbiology* **101**, 713–723 (2012).

70. Vi´lchez, S. *et al. Proline Catabolism by Pseudomonas Putida: Cloning, Characterization, and Expression of the Put Genes in the Presence of Root Exudates*. *JOURNAL OF BACTERIOLOGY* vol. 182 https://journals.asm.org/journal/jb (2000).

71. Chen, S. *et al.* L-Hydroxyproline and d-Proline Catabolism in Sinorhizobium meliloti. *J Bacteriol* **198**, 1171–1181 (2016).

72. Revelles, O., Espinosa-Urgel, M., Fuhrer, T., Sauer, U. & Ramos, J. L. Multiple and interconnected pathways for l-lysine catabolism in Pseudomonas putida KT2440. *J Bacteriol* **187**, 7500–7510 (2005).

73. Agorsor, I. D. K., Kagel, B. T. & Danna, C. H. The Arabidopsis LHT1 Amino Acid Transporter Contributes to Pseudomonas simiae-Mediated Plant Growth Promotion by Modulating Bacterial Metabolism in the Rhizosphere. *Plants (Basel)* **12**, (2023).

74. Matilla, M. A., Espinosa-Urgel, M., Rodríguez-Herva, J. J., Ramos, J. L. & Ramos-González, M. I. Genomic analysis reveals the major driving forces of bacterial life in the rhizosphere. *Genome Biol* **8**, (2007).

75. Manuel, E. U. & Ramos, J. L. Expression of a Pseudomonas putida Aminotransferase Involved in Lysine Catabolism Is Induced in the Rhizosphere. *Appl Environ Microbiol* **67**, 5219–5224 (2001).

76. Kuiper, I., Bloemberg, G. V, Noreen, S., Thomas-Oates, J. E. & Lugtenberg, B. J. J. *Increased Uptake of Putrescine in the Rhizosphere Inhibits Competitive Root Colonization by Pseudomonas Fluorescens Strain WCS365*. vol. 14 (2001).

77. Liu, Z. *et al.* A Genome-Wide Screen Identifies Genes in Rhizosphere-Associated Pseudomonas Required to Evade Plant Defenses. *mBio* **9**, 1–17 (2018).

78. Comai, L. & Kosuge, T. Cloning characterization of iaaM, a virulence determinant of Pseudomonas savastanoi. *J Bacteriol* **149**, 40–46 (1982).

79. Mazzola, M. & White, F. F. A mutation in the indole-3-acetic acid biosynthesis pathway of Pseudomonas syringae pv. syringae affects growth in Phaseolus vulgaris and syringomycin production. *J Bacteriol* **176**, 1374–1382 (1994).

80. Zhang, P. *et al.* The Distribution of Tryptophan-Dependent Indole-3-Acetic Acid Synthesis Pathways in Bacteria Unraveled by Large-Scale Genomic Analysis. *Molecules* **24**, (2019).

81. Barazani, O. & Friedman, J. Is IAA the major root growth factor secreted from plant-growth-mediating bacteria? *J Chem Ecol* **25**, 2397–2406 (1999).

82. Liu, Y. *et al.* Plant-microbe communication enhances auxin biosynthesis by a root-associated bacterium, Bacillus amyloliquefaciens SQR9. *Molecular Plant-Microbe Interactions* **29**, 324–330 (2016).

83. Rädisch, R. *et al.* Metabolism of Aldoximes and Nitriles in Plant-Associated Bacteria and Its Potential in Plant-Bacteria Interactions. *Microorganisms* **10**, 549 (2022).

84. Tullio, L. D. *et al.* Revealing the roles of y4wF and tidC genes in Rhizobium tropici CIAT 899: biosynthesis of indolic compounds and impact on symbiotic properties. *Arch Microbiol* **201**, 171–183 (2019).

85. Malhotra, M. & Srivastava, S. Organization of the ipdC region regulates IAA levels in different Azospirillum brasilense strains: molecular and functional analysis of ipdC in strain SM. *Environ Microbiol* **10**, 1365–1373 (2008).

86. van Puyvelde, S. *et al.* Transcriptome Analysis of the Rhizosphere Bacterium Azospirillum brasilense Reveals an Extensive Auxin Response. *Microb Ecol* **61**, 723–728 (2011).

87. Nascimento, F. X., Glick, B. R. & Rossi, M. J. Multiple plant hormone catabolism activities: an adaptation to a plant-associated lifestyle by Achromobacter spp. *Environ Microbiol Rep* **13**, 533–539 (2021).

88. Zúñiga, A. *et al.* Quorum sensing and indole-3-acetic acid degradation play a role in colonization and plant growth promotion of Arabidopsis thaliana by Burkholderia phytofirmans PsJN. *Mol Plant Microbe Interact* **26**, 546–553 (2013).

89. Greenhut, I. V., Slezak, B. L. & Leveau, J. H. J. iac Gene Expression in the Indole-3-Acetic Acid-Degrading Soil Bacterium Enterobacter soli LF7. *Appl Environ Microbiol* **84**, (2018).

90. Conway, J. M. *et al.* Diverse MarR bacterial regulators of auxin catabolism in the plant microbiome Check for updates. *Nature Microbiology |* **7**, 1817–1833 (2022).

91. Finkel, O. M. *et al.* A single bacterial genus maintains root growth in a complex microbiome. *Nature 2020 587:7832* **587**, 103–108 (2020).

92. Lebeis, S. L. *et al.* Salicylic acid modulates colonization of the root microbiome by specific bacterial taxa. *Science (1979)* **349**, 860–864 (2015).

93. Doornbos, R. F., Geraats, B. P. J., Kuramae, E. E., Van Loon, L. C. & Bakker, P. A. H. M. Effects of jasmonic acid, ethylene, and salicylic acid signaling on the rhizosphere bacterial community of Arabidopsis thaliana. *Mol Plant Microbe Interact* **24**, 395–407 (2011).

94. Lowe-Power, T. M. *et al.* Degradation of the Plant Defense Signal Salicylic Acid Protects Ralstonia solanacearum from Toxicity and Enhances Virulence on Tobacco. *mBio* **7**, (2016).

95. Mark, G. L. *et al. Transcriptome Profiling of Bacterial Responses to Root Exudates Identifies Genes Involved in Microbe-Plant Interactions*. www.genome. (2005).

96. Volkova, O. V. *et al.* Effects of Naphthalene Degradative Plasmids on the Physiological Characteristics of Rhizosphere Bacteria of the Genus Pseudomonas. *Applied Biochemistry and Microbiology 2005 41:5* **41**, 460–464 (2005).
