# Supplementary material for: Predicting rhizosphere-competence-related catabolic gene clusters in plant-associated bacteria with rhizoSMASH
Source: Nat Commun. 2025 Sep 25;16:8400. doi: 10.1038/s41467-025-63526-8 (PMC12462448; doi:10.1038/s41467-025-63526-8)
Supplement: Supplementary file 2 — Description of Additional Supplementary Files [file 41467_2025_63526_MOESM2_ESM.pdf]

Description of Additional Supplementary Files

Supplementary Data 1

Supporting data tables for: 1. Profile hidden Markov models used for building rhizoSMASH, 2. Source and description for genomes in the BARS collection, 3-6. rCGC presence/absence profiles.
