# Supplementary material for: Predicting rhizosphere-competence-related catabolic gene clusters in plant-associated bacteria with rhizoSMASH
Source: Nat Commun. 2025 Sep 25;16:8400. doi: 10.1038/s41467-025-63526-8 (PMC12462448; doi:10.1038/s41467-025-63526-8)
Supplement: Supplementary file 4 — Reporting Summary [file 41467_2025_63526_MOESM4_ESM.pdf]

nature portfolio

Corresponding author(s): Medema, Song

Last updated by author(s): Mar 28, 2025

Reporting Summary

Nature Portfolio wishes to improve the reproducibility of the work that we publish. This form provides structure for consistency and transparency in reporting. For further information on Nature Portfolio policies, see our Editorial Policies and the Editorial Policy Checklist.

Statistics

For all statistical analyses, confirm that the following items are present in the figure legend, table legend, main text, or Methods section.

n/a	Confirmed	
☐	☒	The exact sample size (*n*) for each experimental group/condition, given as a discrete number and unit of measurement
☐	☒	A statement on whether measurements were taken from distinct samples or whether the same sample was measured repeatedly
☐	☒	The statistical test(s) used AND whether they are one- or two-sided<br>*Only common tests should be described solely by name; describe more complex techniques in the Methods section.*
☐	☒	A description of all covariates tested
☐	☒	A description of any assumptions or corrections, such as tests of normality and adjustment for multiple comparisons
☐	☒	A full description of the statistical parameters including central tendency (e.g. means) or other basic estimates (e.g. regression coefficient) AND variation (e.g. standard deviation) or associated estimates of uncertainty (e.g. confidence intervals)
☐	☒	For null hypothesis testing, the test statistic (e.g. *F*, *t*, *r*) with confidence intervals, effect sizes, degrees of freedom and *P* value noted<br>*Give P values as exact values whenever suitable.*
☒	☐	For Bayesian analysis, information on the choice of priors and Markov chain Monte Carlo settings
☐	☒	For hierarchical and complex designs, identification of the appropriate level for tests and full reporting of outcomes
☒	☐	Estimates of effect sizes (e.g. Cohen's *d*, Pearson's *r*), indicating how they were calculated

Our web collection on statistics for biologists contains articles on many of the points above.

Software and code

Policy information about availability of computer code

Data collection	Genome assemblies were downloaded from GenBank using custom code at this repository (https://git.wur.nl/rhizosmash/rhizosmash-case-studies) or from JGI IMAGE manually under GOLD study ID Gs0017561 (for the second case study). Within which, RhizoBase/download-genomes.sh downloads all genome assemblies for the BARS genome collection mentioned in the manuscript, and pseudomonas-60-isolates/script-collect-genomes.py downloads genome assemblies for the first case study.<br>The custom software rhizoSMASH (working version 0.3) was used to generate the rCGC presence/absence information. The source code and the instruction for installation of rhizoSMASH can be found at this repository (https://git.wur.nl/rhizosmash/rhizosmash).
Data analysis	Scripts for reproducing all data analyses in the manuscript can be found at this repository (https://git.wur.nl/rhizosmash/rhizosmash-case-studies), which are:<br>* gene-cluster-distribution/visualize-taxa-distribution.Rmd<br>* gene-cluster-distribution/visualize-burkholderia-chromids.Rmd<br>* pseudomonas-60-isolates/analysis-random-forest.Rmd<br>* avena-rhizobacteria-39-isolates/analysis-random-forest.Rmd

For manuscripts utilizing custom algorithms or software that are central to the research but not yet described in published literature, software must be made available to editors and reviewers. We strongly encourage code deposition in a community repository (e.g. GitHub). See the Nature Portfolio guidelines for submitting code & software for further information.

Data

Policy information about availability of data

All manuscripts must include a data availability statement. This statement should provide the following information, where applicable:

- Accession codes, unique identifiers, or web links for publicly available datasets
- A description of any restrictions on data availability
- For clinical datasets or third party data, please ensure that the statement adheres to our policy

Our analyses were based on public datasets, accessions to these data have been described in the main text and supplementary information of our manuscript.

Research involving human participants, their data, or biological material

Policy information about studies with human participants or human data. See also policy information about sex, gender (identity/presentation), and sexual orientation and race, ethnicity and racism.

Reporting on sex and gender	not applicable.
Reporting on race, ethnicity, or other socially relevant groupings	not applicable.
Population characteristics	not applicable.
Recruitment	not applicable.
Ethics oversight	not applicable.

Note that full information on the approval of the study protocol must also be provided in the manuscript.

Field-specific reporting

Please select the one below that is the best fit for your research. If you are not sure, read the appropriate sections before making your selection.

☐ Life sciences ☐ Behavioural & social sciences ☒ Ecological, evolutionary & environmental sciences

For a reference copy of the document with all sections, see nature.com/documents/nr-reporting-summary-flat.pdf

Ecological, evolutionary & environmental sciences study design

All studies must disclose on these points even when the disclosure is negative.

Study description	This study investigated the rCGC presence/absence profiles from various soil- or rhizoshere-dwelling bacterial strains. We analyzed these profiles with different aspects and scopes. In the later part of our study, we also utilized two datasets from other published studies, for which the properties were described in the materials and methods section and can be found in their original publications.
Research sample	We customized the BActeria in the Rhizosphere and Soil (BARS) collection of genome assemblies, which has been described in the materials and methods section and the supplementary information.
Sampling strategy	not applicable.
Data collection	not applicable.
Timing and spatial scale	not applicable.
Data exclusions	For family-level study, we excluded bacterial families with less than 8 genomes in the BARS collection.
Reproducibility	The BARS collection can be reconstructed with the script at this repository (https://git.wur.nl/rhizosmash/rhizosmash-case-studies/-/blob/main/RhizoBase/download-genomes.sh). The data analysis procedure can be reproduced with the R markdown document at this repository (https://git.wur.nl/rhizosmash/rhizosmash-case-studies/-/blob/main/gene-cluster-distribution/visualize-rhizobase-genome-distance.Rmd).
Randomization	not applicable.
Blinding	not applicable.

Did the study involve field work? ☐ Yes ☒ No

Reporting for specific materials, systems and methods

We require information from authors about some types of materials, experimental systems and methods used in many studies. Here, indicate whether each material, system or method listed is relevant to your study. If you are not sure if a list item applies to your research, read the appropriate section before selecting a response.

Materials & experimental systems

n/a	Involved in the study
☒	☐ Antibodies
☒	☐ Eukaryotic cell lines
☒	☐ Palaeontology and archaeology
☒	☐ Animals and other organisms
☒	☐ Clinical data
☒	☐ Dual use research of concern
☒	☐ Plants

Methods

n/a	Involved in the study
☒	☐ ChIP-seq
☒	☐ Flow cytometry
☒	☐ MRI-based neuroimaging

Plants

Seed stocks	not applicable.
Novel plant genotypes	not applicable.
Authentication	not applicable.
